# Kimchi and *Leuconostoc mesenteroides* DRC 1506 Alleviate Dextran Sulfate Sodium (DSS)-Induced Colitis via Attenuating Inflammatory Responses

**DOI:** 10.3390/foods12030584

**Published:** 2023-01-30

**Authors:** Hye-Jung Moon, Suk-Heung Oh, Ki-Bum Park, Youn-Soo Cha

**Affiliations:** 1 Department of Food Science and Human Nutrition, Jeonbuk National University, Jeonju 54896, Republic of Korea; 2 Department of Food & Biotechnology & Woosuk Institute of Smart Convergence Life Care, Woosuk University, Wanju 55338, Republic of Korea; 3 Institute of Kimchi Technology, Daesang Co., Icheon 17384, Republic of Korea; 4 K-Food Research Center, Jeonbuk National University, Jeonju 54896, Republic of Korea

**Keywords:** kimchi, *Leuconostoc mesenteroides*, dextran sulfate sodium, colitis, anti-inflammatory, mucosal barrier

## Abstract

Ulcerative colitis (UC) is caused by inflammation only in the mucosa of the colon, and its incidence is increasing worldwide. The intake of probiotics is known to have a beneficial effect on the development of UC. In this study, we investigated the alleviating effects of kimchi (KC), a fermented food rich in probiotics, and *Leuconostoc mesenteroides* DRC 1506 (DRC) isolated from kimchi on UC. A freeze-dried kimchi suspension and DRC were orally given to mice at a dose of 1 × 10^9^ CFU/day for 3 weeks. Furthermore, 3% dextran sulfate sodium (DSS) in drinking water was given to induce UC. The KC and DRC groups reduced symptoms of colitis, such as disease activity index, decrease in colon length, colon weight-to-length ratio, and pathological damage to the colon caused by DSS treatment. The KC and DRC groups decreased the levels of pro-inflammatory cytokine (TNF-α) and increased anti-inflammatory cytokine (IL-10) in the colon tissues. At the mRNA and protein expression levels in the colon tissue, KC and DRC groups downregulated inflammatory factors and upregulated tight junction-related factors. Therefore, DRC, as well as KC supplementation, are potent in alleviating UC by improving the inflammatory response and mucosal barrier function in the colon.

## 1. Introduction

Inflammatory bowel disease (IBD), including Crohn’s disease and ulcerative colitis (UC), occurs frequently in the West and has become more common in the East in recent decades to the point where it now requires worldwide management [1,2]. A chronic disease characterized by recurrent inflammation in the gastrointestinal tract, IBD’s exact cause is unknown, but genetic susceptibility, the environment, intestinal microbiota, and mucosal immune response are assumed to be contributing factors. An imbalance in intestinal microbiota due to genetic and environmental factors can destroy a mucosal immune system and damage the intestinal barrier [2,3,4]. Among the various manifestations of IBD, UC occurs more frequently than Crohn’s disease and has the characteristic that mucosal inflammation occurs only in the colon [1,2,5]. Cases of UC usually present with bloody stool, abdominal pain, and rectal urgency. Biologics such as mesalamine, anti-tumor necrosis factor (TNF), small-molecule Janus kinase inhibitors, and anti-integrin are used to treat UC. However, side effects such as immunosuppression and the need for surgery after failure to respond to drug treatment have been reported [6,7]. Such drugs are secondary alternatives that block the response of the inflammatory cascade, not primary solutions [8]. Therefore, it is more important to inhibit the development of UC, and to do so, the intestinal microbiota and immune systems need to maintain homeostasis.

Diets reportedly affect intestinal microbial diversity, barrier function, and immune tolerance, resulting in UC progression. Consumption of highly processed foods, refined sugars, and saturated fats exacerbate colon inflammation, whereas fresh fruits, dietary fiber, omega-3 fatty acids, and probiotics improve symptoms of UC [4,8,9,10]. Probiotics such as *Lactobacillus* and *Bifidobacterium* have been shown to enhance barrier function by increasing the production of tight junction (TJ) protein and mucin by interacting with intestinal epithelial cells. They also reduce the inflammatory response by inhibiting various signaling pathways, such as nuclear factor-kappa B (NF-κB) [8,10,11].

Kimchi is a traditional vegetable fermented food in Korea. It is made by fermenting cabbage and radishes with salt, garlic, red pepper powder, and salted fish with lactic acid bacteria (LAB) [12]. Kimchi contains organic acids, vitamins, bacteriocins, dietary fiber, and flavoring compounds, as well as potential probiotics [12,13]. The major LAB genera of kimchi in the fermentation process are *Leconostoc*, *Lactobacillus*, and *Weisella*. The microbial community in kimchi varies depending on the ingredients, the presence or absence of a starter, the ripening period, and temperature. *Leuconostoc* and *Weisella* dominate in the early- and mid-fermentation periods, and *Lactobacillus* dominates during the late fermentation of cabbage kimchi at low temperatures [14,15,16,17,18,19]. Among these strains, the genus *Leuconostoc* was shown to convert fructose to mannitol, which gives kimchi a refreshing taste [12,16]. *Leuconostoc* is a major strain in ripe kimchi and is used primarily as a starter for the promotion of the preferred varieties of commercially produced kimchi [16,20,21]. Various functions of LAB isolated from kimchi have been reported. *Leuconostoc* (*Leu.*) *citreum* is associated with immunomodulatory and anti-arthritis effects; *Lactiplantibacillus (Lpb.) plantarum* (formerly *Lactobacillus plantarum*) LRCC 5273 has anti-hypercholesterolemia effects; *Weissella cibaria* JW15 has anti-inflammatory effects; and *Levilactobacillus (Lev.) brevis* (formerly *Lactobacillus brevis*) OPK-3 has anti-obesity effects [12,13,22,23,24,25,26]. LAB isolated from kimchi *Lactobacillus*, including *Lacticaseibacillus* (*Lcb.*) *paracasei* (formerly *Lactobacillus paracasei)* LS2, *Lev. brevis* G-101, and *Latilactobacillus (Lat.) curvatus* WiKim38, reportedly ameliorated colitis in a dextran sulfate sodium (DSS)-induced mouse model [12,13,27,28,29]. However, few studies have been reported on the protective effects of *Leu. mesenteroides*, a major strain of kimchi, against colitis. In a previous study, DRC isolated from kimchi was reported to have high acid and bile tolerance and mannitol-production ability [30]. The purpose of this study was to observe whether *Leu. mesenteroides* alleviate colitis, as well as to compare the anti-inflammatory effects when *Leu. mesenteroides* was consumed alone or in the form of kimchi.

## 2. Materials and Methods

### 2.1. Culture of Leu. mesenteroides

*Leu. mesenteroides* DRC 1506 (DRC) were provided by Daesang Co., Ltd. (Seoul, Republic of Korea). DRC was subcultured for 24 h at 25 °C in MRS broth (BD Difco, Detroit, MI, USA). The cultured strains were centrifuged (1200× *g*, 5 min, 4 °C) and washed twice with sterile phosphate-buffered saline (PBS). The collected pellets were resuspended using a 20% glycerol solution and then stored at −70 °C until use. To administer to mice, the stored strain was thawed, washed twice with PBS, and then adjusted to a concentration of 5 × 10^9^ colony-forming units (CFU)/mL with PBS. Then 0.2 mL of this suspension (1 × 10^9^ CFU) was given to each mouse every day.

### 2.2. Preparation of Kimchi Samples

Commercial kimchi manufactured by the method of Korean agent No. 10-1809447 was provided by Daesang Co., Ltd. (Seoul, Republic of Korea), and freeze-dried to make a powder [30]. The LAB counts in freeze-dried kimchi were tested using a 10-fold serial dilution with PBS. The diluted solution was plated on MRS agar (BD Difco, Detroit, MI, USA) and incubated for 48 h at 25 °C. The freeze-dried kimchi, in which LAB counts were confirmed, was suspended in PBS, and 1 × 10^9^ CFU/0.2 mL per mouse was administered orally every day.

### 2.3. Experimental Design Inducing Colitis with DSS

**Figure 1:**
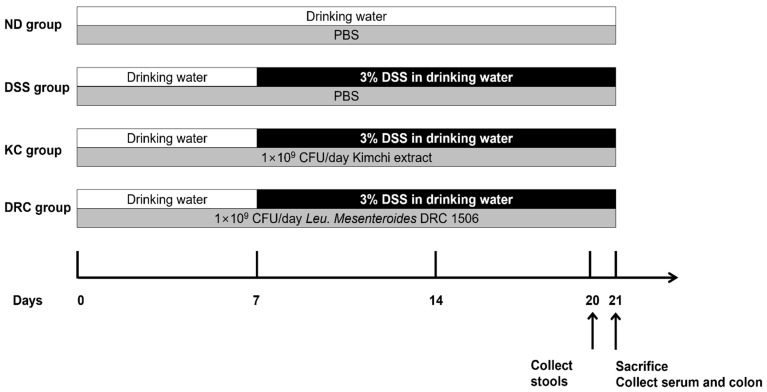
Experimental scheme showing the treatment period of DSS and samples. Samples were treated for 21 days. Except for the ND group, all other groups were given 3% DSS of drinking water starting on the 7th day after the study began. ND, normal group; DSS, DSS-treated group; KC, DSS + 1 × 10^9^ CFU/day/mouse of kimchi group; DRC, DSS + 1 × 10^9^ CFU/day/mouse of *Leu. mesenteroides* DRC 1506 isolated from kimchi.

Four-week-old male C57BL/6J mice were purchased from Dooyeol Biotech (Seoul, Republic of Korea). All mice were provided with a chow diet and maintained at 22 ± 3 °C with a relative humidity of 55 ± 5 °C in a 12 h light/dark cycle. After a 3-week adaptation period, the mice were randomly divided into 4 groups (total *n* = 32, *n* = 8 per group): a normal group (ND), a DSS-treated group, a kimchi-treated (KC) group (administered DSS and kimchi), and a DRC group (administered DSS and *Leu. mesenteroides* DRC 1506). Then 0.2 mL of PBS (ND and DSS groups) or 0.2 mL suspension containing 1 × 10^9^ CFU of LAB (KC and DRC groups) was administered to mice by oral gavage daily for 3 weeks. Access to water and an AIN-93G diet was supplied during all experimental periods. In the second week of the experiment, 3% (*w*/*v*) DSS (36–50 kDa, MP Biomedicals, Solon, OH, USA) in drinking water was provided to all but the ND group to induce colitis (Figure 1). All experiments were approved by the Institutional Animal Care and Use Committees of Jeonbuk National University (approval number JBNU 2021-02).

Feces were collected on the 20th day of the experiment and stored at −70 °C until analysis. At the end of the experiment, mice that had been fasting for 12 h were sacrificed. Blood and colonic tissues were obtained. Blood was centrifuged (1500× *g*, 15 min, 4 °C) to separate serum and placed at −70 °C until analysis. Colon tissue was taken from the caecum to the rectum, the length measured, and the weight measured after removing the feces from the colon. The rectal part of the colon tissue was cut and fixed in a 10% formalin solution. The remaining colon tissue was rapidly frozen with liquid nitrogen and then kept at −70 °C.

### 2.4. Disease Activity Index (DAI)

During the induction of colitis with DSS, body weight, stool consistency, and gross bleeding were checked daily. The DAI score was determined following a procedure described by Mennigen et al. (2009) [31]. Scores were calculated according to the following values: weight loss (0 = none; 1 = 1–5%; 2 = 5–10%; 3 = 10–20%; 4 = >20%), stool consistency (0 = normal; 2 = loose stools; 4 = watery diarrhea), and gross bleeding (0 = none; 2 = occult bleeding; 4 = gross bleeding). The DAI score was expressed as the sum of the body weight loss, the consistency of the stool, and the gross bleeding, divided by 3.

### 2.5. Colonic Histological Damage

Histological damage to colon tissues was confirmed by requesting an examination by the KP&T (Korean Pathology Technical Center, Chungcheongbuk-do, Cheongju, Republic of Korea). Colon tissues fixed in 4% formalin were made into paraffin sections, and hematoxylin and eosin (H&E) staining was performed. Stained slides were verified at 100× and 200× magnification by an Axiophot Zeiss ZI microscope (Carl Zeiss, Gottingen, Germany) at the Center for University-Wide Research Facilities (CURF) at Jeonbuk National University.

### 2.6. Microbial Count in the Feces

A selective medium was used to confirm *Bifidobacterium*, *Enterobacteriaceae (Escherichia coli, Salmonella)*, and *Lactobacillus* in feces [32]. The collected feces were diluted by decimal dilution with sterile PBS. The dilution was dispensed into each medium, spread and cultured, and expressed as log CFU/g. Blood liver agar (BL agar, MBcell, Seoul, Republic of Korea) and desoxycholate agar (MBcell, Seoul, Republic of Korea) were cultured in anaerobic conditions at 37 °C for 1 day and 2 days, respectively. MRS agar (Difco, Detroit, MI, USA) was cultured aerobically at 37 °C for 1 day.

### 2.7. Enzyme-Linked Immunosorbent Assay (ELISA)

In serum and colon, the inflammatory-related cytokines TNF-alpha (TNF-α), interleukin (IL)-1β, IL-6, and IL-10 were analyzed according to manufacturer instructions (R&D systems, Minneapolis, MN, USA). Serum prepared as described in the above method was used. The colon tissue was prepared as follows: A 0.1 g sample of colonic tissue was homogenized on ice with 1% protease inhibitors and 1% phosphatase inhibitor in mL of chilled RIPA lysis buffer. After that, the lysate was centrifuged at 15,000× *g* and 4 °C for 10 min to obtain a supernatant for use in the experiment.

### 2.8. Quantitative Real-Time Polymerase Chain Reactions (qRT-PCR)

Total RNA from colon tissue was extracted with a RNeasy Mini kit (Qiagen, Duesseldorf, Germany) according to the manufacturer’s instructions. The extracted RNA was synthesized into cDNA by reverse transcription using a PrimeScript RT reagent kit (TaKaRa Bio, Otsu, Japan). The synthesized cDNA was amplified by qRT-PCR amplification using SYBR Green and appropriate primers (Table S1). The reaction of qRT-PCR was pre-denaturation at 95 °C for 10 min, and then 40 cycles were performed under the thermocycling conditions for amplification in denaturation (95 °C, 15 s), annealing (60 °C, 20 s), and extension (72 °C, 35 s). The relative mRNA expression of the target genes was calculated using the 2-delta delta Ct method by β-actin as reference. The value was expressed as fold changes with the ND group as control.

### 2.9. Western Blotting

Proteins were extracted from colon tissues using a RIPA buffer (Thermo Scientific, Rockford, IL, USA) with phosphatase inhibitor and protease inhibitor. Total protein concentration was measured with a bicinchoninic acid protein kit (Pierce, Thermo Scientific, Rockford, IL, USA). Proteins were loaded onto SDS–PAGE and electrophoresed with molecular weight size. After transferring them to a polyvinylidene fluoride (PVDF) membrane (Bio-rad, Hercules, CA, USA), they were blocked with a 5% bovine serum albumin solution. The primary antibody was allowed to react overnight at 4 °C and washed with TBST, and the secondary antibody was attached at room temperature for 1 h. After washing with TBST, the membrane with an enhanced chemiluminescence solution (GE Health, Buckinghamshire, UK) was allowed to react and was observed with ChemiDoc (Bio-rad, Hercules, CA, USA). Protein bands were normalized to β-actin and quantified with ND groups using ImageJ software (National Institute of Health, Bethesda, MD, USA). The list of antibodies used in the study is shown in Table S2.

### 2.10. Statistical Analysis

Data were analyzed using the program SPSS 18.0 (SPSS Inc., Armonk, NY, USA). The data for the change in body weight and DAI, gut microbial counts, and levels of protein expression in the colon were the mean ± standard deviation (SD). Data for other parameters were presented as scatter plots with median, minimum, and maximum values (*n* = 6–8 per group). The colon length, the image of H&E staining, and the image of the Western blot were representative photographs of each group. Significant differences among the four groups were analyzed by one-way analysis of variance (ANOVA) with Duncan’s post hoc test, with different letters above the values. The *p*-value of <0.05 was used to determine the statistical significance of all data.

## 3. Results

### 3.1. Effects of Kimchi and DRC on Body Weight and Colitis Symptoms

**Figure 2:**
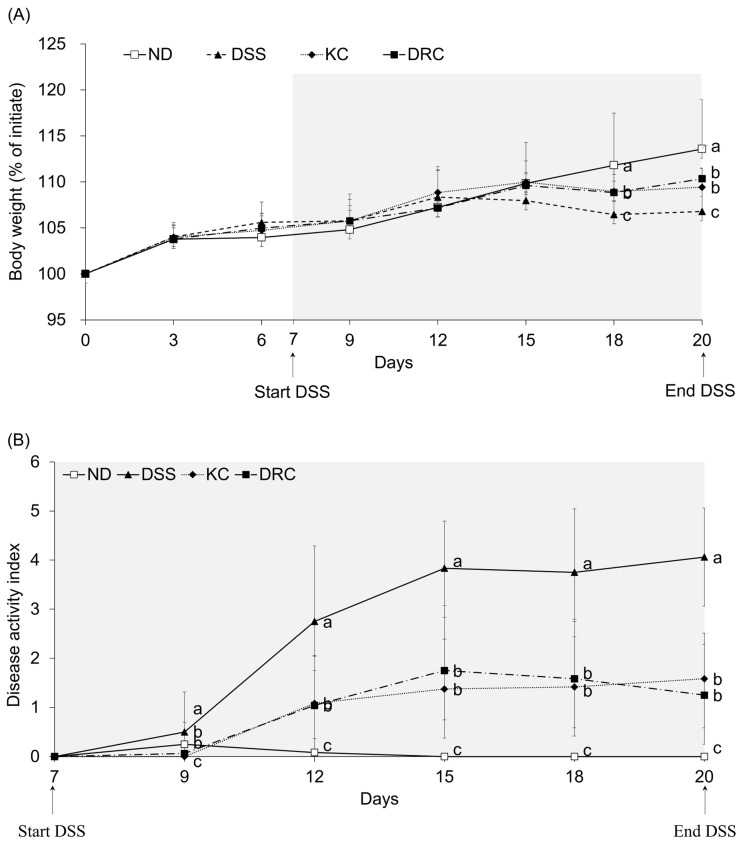
Effects of kimchi and *Leu. mesenteroides* DRC 1506 on changes of body weight (**A**) and disease activity index (DAI) (**B**) during DSS treatment. (**A**) The body weight was measured daily at the same time as the start of the experiment, and (**B**) DAI was evaluated every day after starting DSS drinking. Comparisons among groups were evaluated using one-way ANOVA with Duncan’s post hoc test. Groups with different letters had a significant difference at *p* < 0.05. ND, normal group; DSS, DSS-treated group; KC, DSS + 1 × 10^9^ CFU/day/mouse of kimchi group; DRC, DSS + 1 × 10^9^ CFU/day/mouse of *Leu. mesenteroides* DRC 1506 isolated from kimchi.

In order to examine the effect of restoring colitis, the samples were administered to mice for one week, and colitis was generated with DSS. On the 12th day after inducing colitis with 3% DSS, the DSS groups began to show significant changes in body weight compared with the ND group (Figure 2A and Figure S1). Compared with the DSS group, there began to be a significant difference in the DRC group and KC group on the 12th day after drinking DSS. The final body weight was significantly higher in the KC and DRC groups than in the DSS group. As for DAI, from the 2nd day of DSS administration, all experimental groups showed significant differences compared with the DSS group (Figure 2B). At the end of the experiment, the DAI scores were significantly lower in all experimental groups compared with the DSS group. Therefore, the KC and DRC were found to relieve symptoms of colitis induced by DSS.

### 3.2. Effects of Kimchi and DRC on Colonic Tissue Damage

**Figure 3:**
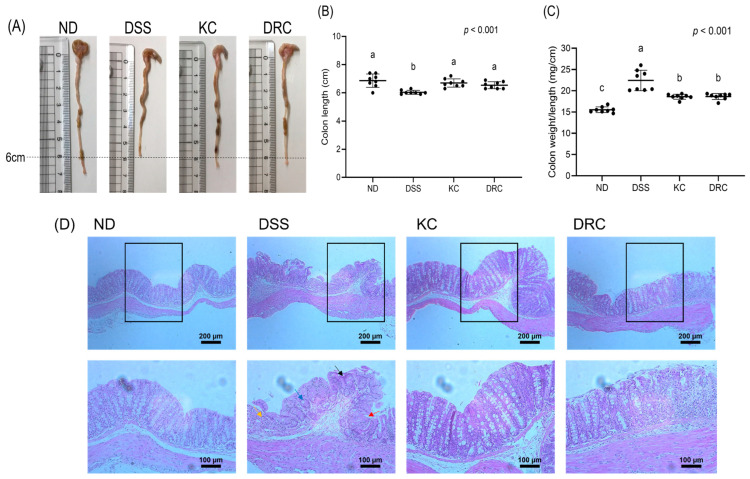
Effects of kimchi and *Leu. mesenteroides* DRC 1506 on colon length (**A**,**B**), colon weight/length (**C**), and histopathology (**D**). (**A**) Representative photographs of colon tissue in each group. (**B**,**C**) The colon length and colon weight-to-length ratio are presented as scatter plots with median, minimum and maximum values (*n* = 8 mice per group). Comparisons among groups were evaluated using one-way ANOVA with Duncan’s post hoc test. Groups with different letters had a significant difference at *p* < 0.05. (**D**) Representative images of H&E staining of colonic sections in each group. Three histological sections were analyzed per animal, with *n* = 3 mice per group. Arrows represent the following histological lesions: crypt destruction (black), reduced goblet cells (blue), inflammatory cell infiltration (yellow), and colonic mucosal erosion (red). Scale bar = 100 μm and 200 μm. ND, normal group; DSS, DSS-treated group; KC, DSS + 1 × 10^9^ CFU/day/mouse of kimchi group; DRC, DSS + 1 × 10^9^ CFU/day/mouse of *Leu. mesenteroides* DRC 1506 isolated from kimchi.

The colon length of the DSS group was significantly reduced compared with the ND group, confirming that the colon tissue was damaged (Figure 3A,B). The KC and DRC groups not only showed significantly higher colon lengths compared with the DSS group, but lengths similar to those of the ND group. The weight per colon length was significantly higher in the DSS group compared with the ND group (Figure 3C). In the KC and DRC groups, the value of the weight per colon length was significantly lower than in the DSS group.

Observations of histopathological changes in the colon revealed no damage to the crypt, mucosal structure, and goblet cells in the ND group (Figure 3D). Compared with the ND group, the DSS group experienced crypt destruction, reduced goblet cells, inflammatory cell infiltration, and colonic mucosal erosion. The KC and DRC groups were associated with improvements in the pathological changes caused by DSS. In particular, the KC group appeared to alleviate the decrease in goblet cells and inflammatory cell infiltration compared with the DRC group.

### 3.3. Effects of Kimchi and DRC on Some Gut Microbial Counts

The number of viable cells of *Bifidobacterium*, *Lactobacillus*, and *Enterobacteriaceae* in the feces was counted to confirm changes in the composition of the gut microbiota (Table 1). For *Bifidobacterium*, *Lactobacillus*, and *Enterobacteriaceae*, the number of viable cells in the DSS group was significantly higher than in the ND group. Compared with the DSS group, *Enterobacteriaceae* was significantly lowered in the KC group, and there was no significant difference in the DRC group, but a decreasing tendency was observed. For *Bifidobacterium* and *Lactobacillus*, no significant difference between the experimental group and the DSS group was seen. Therefore, KC and DRC treatment were shown to have more effect on *Enterobacteriaceae* than on *Bifidobacterium* and *Lactobacillus*.

### 3.4. Effects of Kimchi and DRC on Inflammation-Related Cytokines in Serum and the Colon

**Figure 4:**
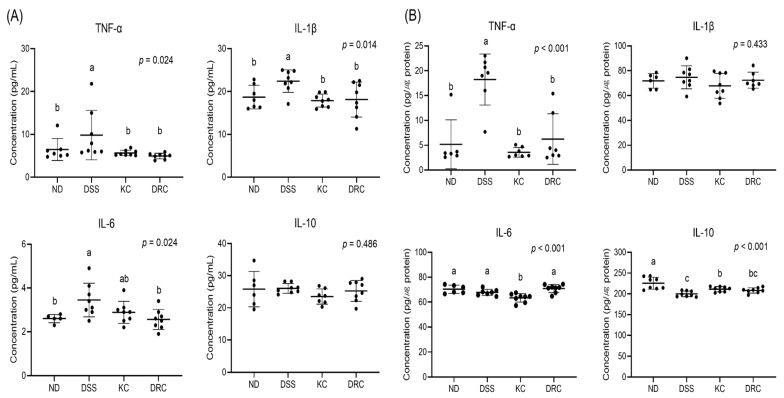
Effects of kimchi and *Leu. mesenteroides* DRC 1506 on pro-inflammatory and anti-inflammatory cytokines in serum (**A**) and colon tissue (**B**). Data values are expressed as scatter plots with median, minimum, and maximum values (*n* = 5–8 per group). Comparisons among groups were evaluated using one-way ANOVA with Duncan’s post hoc test. Groups with different letters had a significant difference at *p* < 0.05. ND, normal group; DSS, DSS-treated group; KC, DSS + 1 × 10^9^ CFU/day/mouse of kimchi group; DRC, DSS + 1 × 10^9^ CFU/day/mouse of *Leu. mesenteroides* DRC 1506 isolated from kimchi.

Cytokines related to inflammation were tested in serum and the colon. In serum TNF-α, IL-1β, and IL-6, the DSS group showed significantly higher values compared to the ND group, whereas no significant difference in IL-10 was seen. The levels of TNF-α and IL-1 β were significantly lower in the KC and DRC groups compared with the DSS group (Figure 4A). In colon tissue, the TNF-α value was significantly lower in the ND, KC, and DRC groups compared with the DSS group. However, IL-10 levels in the colon were significantly higher in the ND and KC groups compared with the DSS group. No significant difference in IL-1β levels of the colon was recorded for any groups (Figure 4B).

### 3.5. Effects of Kimchi and DRC on the Expression of Genes and Proteins Related to the Inflammatory Response in the Colon

**Figure 5:**
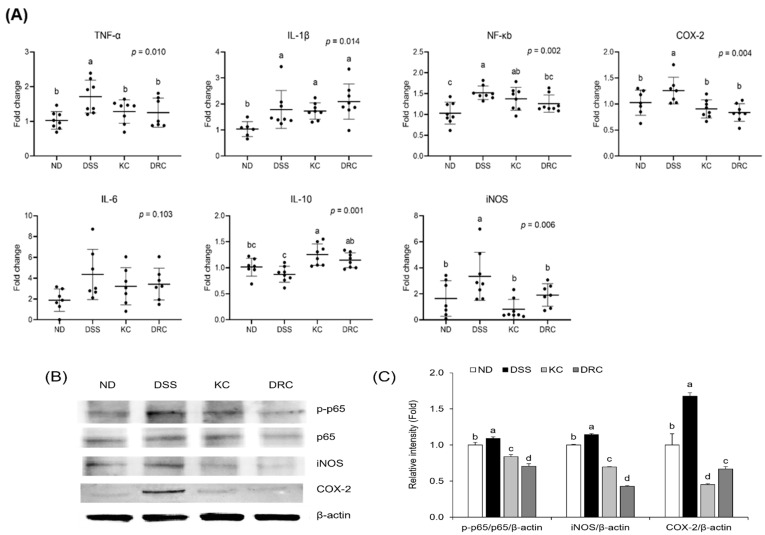
Effects of kimchi and *Leu. mesenteroides* DRC 1506 on the expression of mRNA (**A**) and protein (**B**,**C**) related to the pro-inflammatory factors in colon tissue. (**A**) The relative mRNA expression of the target genes was quantified and normalized by β-actin. The value was expressed as fold changes with the ND group as the control. Data values are expressed as scatter plots with median, minimum, and maximum values (*n* = 6–8 per group). Comparisons among groups were evaluated using one-way ANOVA with Duncan’s post hoc test. Groups with different letters had a significant difference at *p* < 0.05. (**B**) Representative image by Western blot. (**C**) The relative expression level of protein was quantified by Image J. The relative expression levels of protein were quantified and normalized by β-actin. The value was expressed as fold changes with the ND group as the control. Values were presented as bar graphs with means ± SD (*n* = 6 per group). Comparisons among groups were analyzed by one-way ANOVA with Duncan’s post hoc test. Groups with different letters had a significant difference at *p* < 0.05. The presented data were from one representative experiment out of three. ND, normal group; DSS, DSS-treated group; KC, DSS + 1 × 10^9^ CFU/day/mouse of kimchi group; DRC, DSS + 1 × 10^9^ CFU/day/mouse of *Leu. mesenteroides* DRC 1506 isolated from kimchi.

Gene expression related to the inflammatory response was found in colon tissue (Figure 5A). In all indicators except IL-6, the ND group exhibited a significant difference compared with the DSS group. The KC group had significantly lower values of TNF-α, cyclooxygenase-2 (COX-2), and inducible nitric oxide synthase (iNOS) than the DSS group. The DRC group had significantly lower levels of TNF-α, NF-κB, COX-2, and iNOS compared with the DSS group. For IL-10, an anti-inflammatory cytokine, a significantly higher value was shown in all groups when compared with the DSS group. At the protein expression level, the expression levels of iNOS and COX-2 in the KC group and DRC group were significantly reduced compared to the DSS group. In particular, the DRC group showed the lowest expression level of phospho-p65 (p-p65) (Figure 5B,C).

### 3.6. Effects of Kimchi and DRC on the Expression of Genes and Proteins Related to Mucosal Barrier Function in the Colon

**Figure 6:**
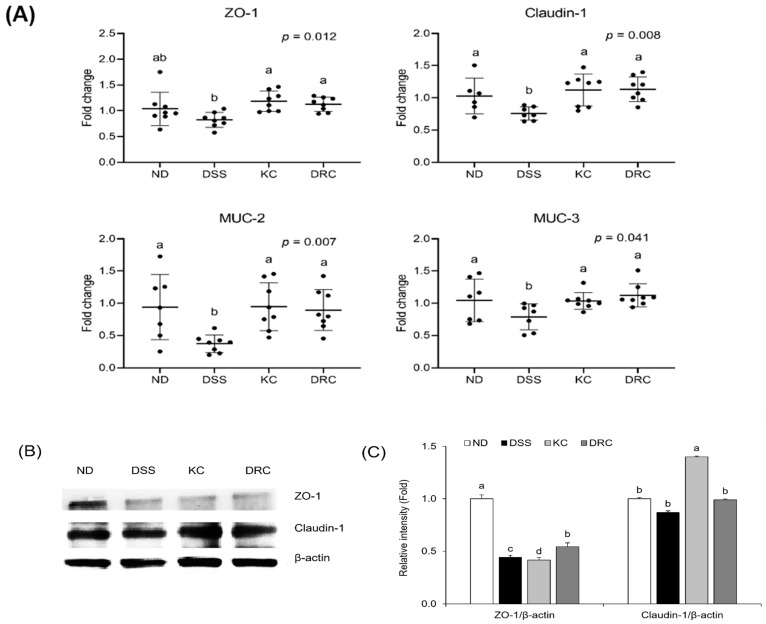
Effects of kimchi and *Leu. mesenteroides* DRC 1506 on the expression of mRNA (**A**) and protein (**B**,**C**) of tight junction protein and mucin. (**A**) The relative mRNA expression of the target genes was quantified and normalized by β-actin. The value was expressed as fold changes, with the ND group as the control. Data values are expressed as scatter plots with median, minimum, and maximum values (*n* = 6–8 per group). Comparisons among groups were evaluated using one-way ANOVA with Duncan’s post hoc test. Groups with different letters had a significant difference at *p* < 0.05. (**B**) Representative image by Western blot. (**C**) The relative expression level of protein was quantified by Image J. The relative expression levels of protein were quantified and normalized by β-actin. The value was expressed as fold changes with the ND group as the control. Values were presented as bar graphs with means ± SD (*n* = 6 per group). Comparisons among groups were analyzed by one-way ANOVA with Duncan’s post hoc test. Groups with different letters had a significant difference at *p* < 0.05. The presented data were from one representative experiment out of three. ND, normal group; DSS, DSS-treated group; KC, DSS + 1 × 10^9^ CFU/day/mouse of kimchi group; DRC, DSS + 1 × 10^9^ CFU/day/mouse of *Leu. mesenteroides* DRC 1506 isolated from kimchi.

The expression of mucosal barrier function-related genes zonula occludens-1 (ZO-1), Claudin-1, mucin (MUC)-2, and MUC-3 was significantly lower in the DSS group than in the ND group. Compared with the DSS group, the KC and DRC groups exhibited significantly higher values in all genes related to mucosal barrier function (Figure 6A). The expression level of the tight junction protein was confirmed using Western blot. As a result, the DRC group significantly increased the expression level of ZO-1 more than the DSS group. On the other hand, the KC group showed a low value at ZO-1 compared to the DSS group, but a significantly higher value at Claudin-1. Therefore, it seems that the KC and DRC groups can improve the barrier function (Figure 6B,C).

## 4. Discussion

The human intestine is directly connected to the external environment and easily exposed to external antigens. To protect the body from external antigens, intestinal mucosal tissue is known to interact with gut microorganisms to maintain the immune system [3]. The exact cause of UC is unclear, but it is known that a genetically susceptible host interacts with environmental factors, destroying the intestinal epithelial barrier, allowing bacterial or bacterial antigens into the mucosa, breaking the intestinal immune system’s homeostasis, and causing intestinal inflammatory reactions [10]. Probiotics and fermented foods containing them have been known to increase beneficial bacteria among gut microbes, protect intestinal barriers, and reduce intestinal inflammation [11,28,31,33,34,35,36,37]. Kimchi, a fermented food, is also rich in probiotics, so it was expected to have a beneficial effect against colitis [13,38,39,40]. Hence, this study attempted to investigate the protective effect of not only kimchi but also LAB isolated from kimchi (*Leu. mesenteroides* DRC 1506) on colitis.

Exposure to DSS has been reported to cause mucosal inflammation by changing the microbial balance and destroying intestinal barrier function and the mucosa [41]. The resulting symptoms of colitis were a decrease in colon length and an increase in the colon weight/length ratio due to weight loss, bloody stool, diarrhea, and inflammatory progression [41,42]. As a result of inducing colitis with DSS while consuming kimchi and DRC, the weight loss, DAI score, colon length, and colonic weight/length ratio were significantly diminished compared with the DSS group. In addition, 10^9^ CFU/day of kimchi and DRC were provided to mice, which were found to ameliorate symptoms of colitis. Previous studies have reported that 10^9^ CFU/mL of *Lat. curvatus* WiKim38, *Lev. brevis* G-101, *Lcb. paracasei* LS2, and *Lpb. plantarum* AR113 improved symptoms produced by DSS, and our findings were similar [27,28,29,43].

Intestinal barrier defense mechanisms that maintain host health include microbial, mucosal, epithelial, and immune-system varieties [44]. The effects of kimchi and DRC on alterations in the intestinal barrier were observed using histology and changes in the gut microbiota during DSS-induced colitis. As revealed by histology exams, the DSS group experienced the destruction of the intestinal mucosal structure, goblet cell reduction, and inflammatory infiltration. However, in the KC and DRC groups, this intestinal damage was alleviated. Probiotics have been shown to strengthen epithelial walls, increase intestinal mucosal adhesion, and regulate the immune system [11]. *Lpb. plantarum* AR113, mixed probiotics (*Limosilactobacillus (Lim.) reuteri* (formerly *Lactobacillus reuteri*) RAM0101, *Bacillus (Ba.) coagulans* RAM1202, *Bifidobacterium (B.) longum* CICC6197, *Clostridium (C.) butyricum* RAM0216), or fermented food containing probiotics have been shown to relieve DSS-induced colitis [43,45,46]. In our results, the intake of kimchi and DRC seems to alleviate intestinal damage, similar to the function of probiotics in previous studies.

An imbalance of gut microbiota is known to cause inflammation by destroying intestinal epithelial cells, increasing intestinal permeability, and destroying mucous membranes [3,10]. In previous studies, the IBD pathogenesis showed a reduction in gut microbial diversity, and the populations of the family *Enterobacteriaceae* (*E. coli*, *Salmonella*, *Shigella)* in the intestine were found to increase in UC [10,47]. In another study that induced UC with DSS, the CFU of *E. coli* belonging to the family *Enterobacteriaceae* in UC mice had considerably greater amounts than that of normal mice [48]. In another animal model that demonstrated the effect of *B. lactis*, *Lcb. casei* (*formerly Lactobacillus casei*), and *Lactobacillus (Lb.*) *acidophilus* on colitis, the control group showed no significant differences in *Bifidobacterium* compared with the sample treatment group but significantly decreased levels in *Lactobacillus* [49]. Our data showed that the KC group experienced no change in *Lactobacillus* and *Bifidobacterium*, but *Enterobacteriaceae* decreased significantly, which is assumed to reduce pathogens. Thus, kimchi seems to have a beneficial effect on the restoration of UC by inhibiting the growth of harmful bacteria in the colon. However, since it has been known that numerous microorganisms exist in the colon, further studies will be needed on the relative abundance of gut microbiota and whether kimchi and DRC intake are related to the development of colitis.

Exposure to DSS damages the intestinal epithelial barrier, allowing intestinal pathogens to enter the intestine, triggering the activation of immune cells such as intestinal macrophages, and thereby producing an inflammatory response [41]. TNF-α, IL-1β, and IL-6 are major pro-inflammation cytokines that play important roles in the development of IBD in inflammation [44], whereas IL-10 is known as an anti-inflammatory cytokine secreted from regulatory T cells that have been shown to diminish inflammation in IBD when expressed at high levels [50]. The levels of TNF-α, IL-1β, and IL-6 in the serum were significantly lower in the KC and DRC groups compared with the DSS groups. Moreover, the KC and DRC groups were found to significantly reduce TNF-α in the colon when compared with the DSS group. IL-10 was found to be increased in the KC group in the colon. NF-κB regulates the expression of pro-inflammatory cytokine-related genes, such as TNF-α, IL-6, and IL-1β [51]. In addition, stimulation with TNF-α and LPS in macrophages is known to increase the expression of COX-2 and iNOS by activating NF-kB, exacerbating inflammation [32,52]. In the kimchi and DRC-treated groups, gene expression levels in the colon showed significant differences in all indicators except for IL-1β and IL-6. In the expression of proteins in the colon, kimchi and DRC were shown to inhibit COX-2 and iNOS. In particular, DRC inhibited the activity of NF-κB, known as a key transcription factor in pro-inflammatory responses. Intestinal epithelial permeability and homeostasis are crucially maintained by the intestinal epithelial barrier. TJ is a protein complex composed of Occludin, ZO-1, Claudins, and junctional adhesion molecules located at the end of the outer membrane of intestinal epithelial cells and plays an important role in regulating intestinal epithelial cells [41]. The mucosal layer protecting the intestinal epithelium is maintained by secretory (MUC-2) and membrane-bound (MUC-3) mucins produced by goblet cells [53]. In our results, KC and DRC were found to significantly increase the expression of the TJ proteins Claudin-1 and ZO-1, respectively. The administration of 10^8^ CFU/mL of *Lb. sakei* K040706 isolated from soybean paste has been reported to inhibit pro-inflammatory factors such as nitric oxide (NO), TNF-α, IL-1β, and IL-6 and reduce inflammatory cell infiltration, thereby weakening the severity of colitis caused by DSS in mice [54]. The KC and DRC groups were shown to inhibit goblet cell destruction and decrease cell infiltration in histology. Similarly, they were also found to improve intestinal epithelial barrier and mucosal protection and inflammation-related factors at the gene and protein expression level. Yan et al. (2019) reported that the family unclassified *Enterobacteriaceae* had a positive correlation with pro-inflammatory cytokines and a negative correlation with TJ proteins and MUC-2 [55]. Likewise, the administration of KC and DRC appeared to reduce *Enterobacteriaceae*, consequently maintaining anti-inflammatory and mucosal barrier activities via reduced TNF-α expression and release, and enhanced TJ protein and MUC-2 expression. In particular, KC showed a tendency to decrease *Enterobacteriaceae* more than did DRC. Kimchi has been documented to be rich in dietary fiber, vitamins, and minerals, as well as probiotics, and these ingredients were associated with reduced levels of harmful bacteria [9,13]. It has also been reported that dietary fiber can produce short-chain fatty acids by fermenting symbiotic bacteria. Short-chain fatty acids are known to lower intestinal pH by suppressing the proliferation of harmful bacteria, maintaining mucosal barrier function, and controlling immune function by using them as an energy source for all epithelial cells [13]. Dietary fiber in kimchi is also thought to be involved in intestinal barrier function and immune regulation, so further research on this is needed. Moreover, kimchi contains not only DRC but also various LABs [15]. A study of the probiotics-alone intake group and four kinds of mixed probiotics (*Lim. reuteri*, *Ba. coagulans*, *B. longum*, *C. butyricum*) showed that mixed probiotics were more effective in alleviating colitis [46]. It is expected that the synergistic effect of various LABs in kimchi used in this study will also affect the alleviation of colitis. Therefore, further studies are needed to investigate the effects of other components in kimchi, such as dietary fiber and mixed lactic acid bacteria, on colitis.

## 5. Conclusions

The anti-inflammatory and beneficial effects on the intestinal mucosal function of kimchi and DRC isolated from kimchi were confirmed when colitis was induced with DSS. KC and DRC groups downregulated the expression of genes and proteins related to pro-inflammatory cytokines (TNF-α, IL-6, and IL-1β) and inductive enzymes including COX-2 and iNOS. KC and DRC were found to reduce NF-κB activity and affect the expression of inflammatory cytokines downstream. In particular, KC increased the level of anti-inflammatory cytokines (IL-10) in the colon. KC and DRC have been shown to alleviate damage to the intestinal mucosal barrier by raising the expression of TJ proteins and mucin-producing genes that maintain the intestinal barrier through the reduction of inflammatory responses and intestinal harmful bacteria. Therefore, it was suggested that kimchi, as well as DRC, the LAB isolated from kimchi, is expected to have the potential to restore colitis.

## Figures and Tables

**Table 1:** Effect of kimchi and *Leu. mesenteroides* DRC 1506 on some gut microbial counts.

	*Bifidobacteria* (log CFU/g)	*Lactobacillus* (log CFU/g)	*Enterobacteriaceae* (log CFU/g)
ND	7.65 ± 0.12 ^b^	6.60 ± 0.76 ^b^	2.85 ± 2.47 ^b^
DSS	9.64 ± 0.25 ^a^	8.77 ± 0.28 ^a^	7.32 ± 0.19 ^a^
KC	9.83 ± 0.18 ^a^	8.99 ± 0.32 ^a^	3.91 ± 1.14 ^b^
DRC	9.50 ± 0.61 ^a^	8.68 ± 1.52 ^a^	5.38 ± 0.73 ^ab^

Data are expressed as the means ± SD (*n* = 4 per group). Comparisons among groups were analyzed by one-way ANOVA with Duncan’s post hoc test. Groups with different letters had a significant difference at *p* < 0.05. ND, normal group; DSS, DSS-treated group; KC, DSS + 1 × 10^9^ CFU/day/mouse of kimchi group; DRC, DSS + 1 × 10^9^ CFU/day/mouse of *Leu. mesenteroides* DRC 1506 isolated from kimchi.

## Data Availability

Data is contained within the article or supplementary material.

## Supplementary Materials

The following supporting information can be downloaded at: https://www.mdpi.com/article/10.3390/foods12030584/s1, Figure S1: Changes in food intake during the experiment periods. Table S1: Primer list used for quantitative real-time PCR. Table S2: Antibodies list used for Western blotting.
