# Supplementary material for: Kimchi and Leuconostoc mesenteroides DRC 1506 Alleviate Dextran Sulfate Sodium (DSS)-Induced Colitis via Attenuating Inflammatory Responses
Source: Foods. 2023 Jan 30;12(3):584. doi: 10.3390/foods12030584 (PMC9914003; doi:10.3390/foods12030584)
Supplement: Supplementary file 1 [file foods-12-00584-s001.zip › foods-2176585-supplementary.pdf]

Supplementary Materials

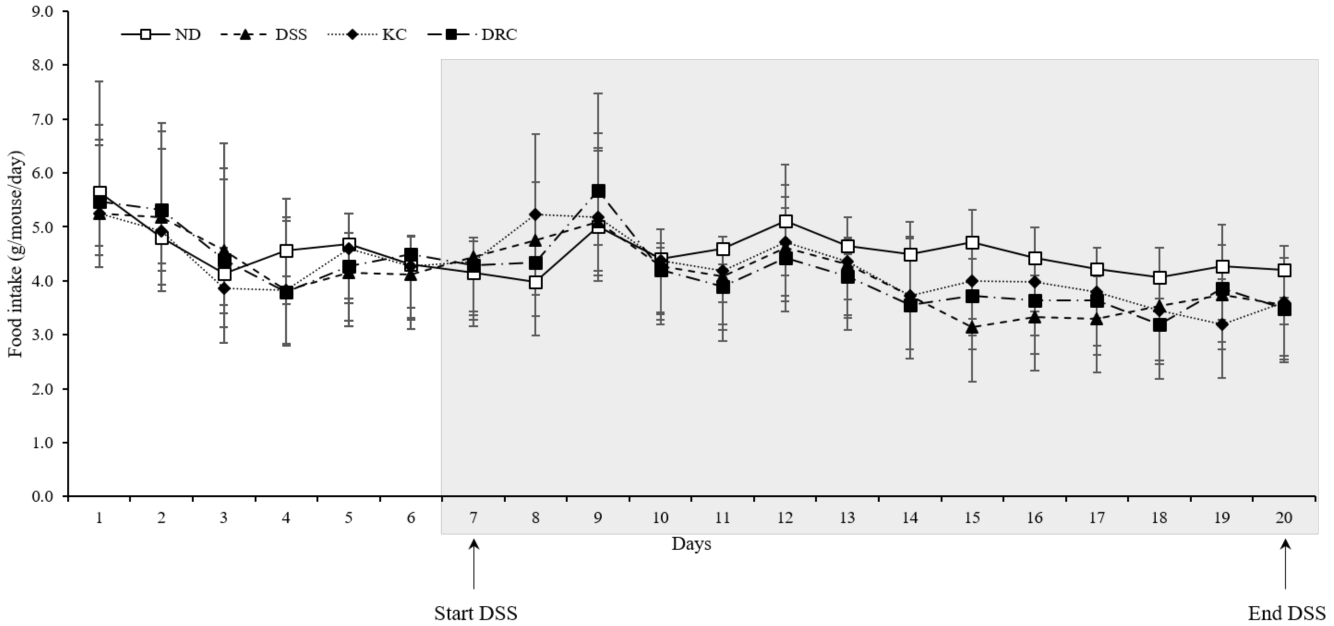

Figure S1. Changes in food intake during the experiment periods. Data were expressed as the means ± SD (*n* = 8 per group). Food intake was measured daily at the same time as the start of the experiment. Comparisons among groups were evaluated using one-way ANOVA with Duncan's post hoc test. Groups with different letters had a significant difference at $p < 0.05$. Blank, no significance. ND, Normal group; DSS, DSS-treated group; KC, DSS + 1×10^9 CFU/day/mouse of kimchi group; DRC, DSS + 1×10^9 CFU/day/mouse of *Leu. mesenteroides* DRC 1506 isolated from kimchi.

Table S1. Primer list used for quantitative real-time PCR

No.	Target gene		Primer sequence 5'→3'
1	TNF-α	F:	5′-ACG GCA TGG ATC TCA AAG AC-3′
		R:	5′-GTG GGT GAG GAG CAC GTA GT-3′
2	IL-1β	F:	5′-GAC CTT CCA GGA TGA GGA CA-3′
		R:	5′-AGC TCA TAT GGG TCC GAC AG-3′
3	IL-6	F:	5′-AAC GAT GAT GCA CTT GCA GA-3′
		R:	5′-GAG CAT TGG AAA TTG GGG TA-3′
4	IL-10	F:	5′-TAC CTG GTA GAA GTG ATG CC-3′
		R:	5′-CAT CAT GTA TGC TTC TAT GC-3′
5	NF-κb	F:	5′-GAA GTG AGA GAG TGA GCG AGA GAG-3′
		R:	5′-CGG GTG GCG AAA CCT CCT C-3′
6	COX-2	F:	5′-GTG GAA AAA CCT CGT CCA GA-3′
		R:	5′-TGA TGG TGG CTG TTT TGG TA-3′
7	iNOS	F:	5′-ATG TCC GAA GCA AAC ATC AC-3′
		R:	5′-TAA TGT CCA GGA AGT AGG TG-3′
8	ZO-1	F:	5′-ACC CGA AAC TGA TGC TGT GGA TAG-3′
		R:	5′-AAA TGG CCG GGC AGA ACT TGT GTA-3′
9	Claudin-1	F:	5′-TCT ACG AGG GAC TGT GGA TG-3′
		R:	5′-TCA GAT TCA GCA AGG AGT CG-3′
10	MUC-2	F:	5′-GCT GCT CAT TGA GAA GAA CGA TGC-3′
		R:	5′-CTC TCC AGG TAC ACC ATG TTA CCA GG-3′
11	MUC-3	F:	5′-CCA CCA CTG TTG AAG TCA CAA-3′
		R:	5′-CAG AAC CCT CCG TTC ATA CAA-3′
12	β-actin	F:	5′-CTG TGC CCA TCT ACG AGG GCT AT-3′
		R:	5′-TTT GAT GTC ACG CAC GAT TTC C-3′

F, forward; R, reverse; TNF-α, tumor necrosis factor-α; IL, interleukin; NF-κb, nuclear factor kappa-light-chain-enhancer of activated B cells; COX-2, cyclooxygenase-2; iNOS, inducible nitric oxide synthase; ZO-1, zonula occludens-1; MUC, mucin.

Table S2. Antibodies list used for Western blotting

Type	Antigen	Origin	Dilution	Catalog number	Manufacturer
primary antibody	phospho-NF-κb p65 (p-p65)	rabbit	1:1000	3033	Cell Signaling Technology
	NF-κb p65 (p65)	rabbit	1:1000	4764	
	iNOS	rabbit	1:1000	13120	
	COX-2	rabbit	1:1000	12282	
	ZO-1	rabbit	1:1000	8193	
	Claudin-1	rabbit	1:1000	13255	
	β-actin	mouse	1:1000	12262	
secondary antibody	Anti-mouse IgG HRP-linked	horse	1:2000	7076	
	Anti-rabbit IgG HRP-linked	goat	1:2000	7074	

NF-κb, nuclear factor kappa-light-chain-enhancer of activated B cells; iNOS, inducible nitric oxide synthase; COX-2, cyclooxygenase-2; ZO-1, zonula occludens-1, IgG, Immunoglobulin G; HRP, horseradish peroxidase.
